# The Analysis of the Clinical Course of Acute Pancreatitis in Children—A Single-Center Study

**DOI:** 10.3390/children12121665

**Published:** 2025-12-08

**Authors:** Aleksandra Mroskowiak, Karolina Majewska, Zuzanna Symela, Dominik Rabstein, Urszula Grzybowska-Chlebowczyk, Sabina Więcek

**Affiliations:** 1 Department of Paediatrics, Faculty of Medical Sciences in Katowice, Medical University of Silesia, 40-752 Katowice, Poland; 2 Department of Digestive Tract Surgery, Faculty of Medical Sciences in Katowice, Medical University of Silesia, 40-752 Katowice, Poland; 3 Department of Pediatric Surgery and Urology, University Hospital in Wroclaw, 50-369 Wrocław, Poland; 4 You Clinic Rabstein, 40-702 Katowice, Poland; 5 Gastroenterology and Paediatric Clinic, Upper-Silesian Children’s Health Centre, 40-752 Katowice, Poland

**Keywords:** acute pancreatitis, children, clinical picture, complication

## Abstract

Acute pancreatitis (AP) is a multifactorial, complicated inflammatory process that involves the organ and the tissues around it. In children, the most common causes of acute pancreatitis are abdominal trauma, infections (mostly viruses), systemic diseases, bile duct diseases (anatomical defects and/or gallstones) and genetic mutations. The course of the disease can vary from mild to very severe with life-threatening complications. The aim of this study was to conduct a retrospective analysis of causes, clinical picture, complications and treatment of acute pancreatitis in children. *Materials and methods:* We retrospectively analyzed the history of 57 children hospitalized in the Department of Paediatrics, Medical University of Silesia in Katowice between 2019 and 2022 with diagnosed acute pancreatitis. *Results:* The analysis included 57 children (age 2–18 years, average 11.0 years, 51% boys, 49% girls) with diagnosed acute pancreatitis. The most common causes of acute pancreatitis were biliary (14/57—24.6%), genetic (10/57—17.5%) and anatomical defects (8/57—14%). In 20/57 (35.1%) children, idiopathic acute pancreatitis was diagnosed. The genetically determined causes were the following: SPINK1 mutation in 5/57 (8.7%) children, PRSS1 mutation in 4/57 (7%) patients and CPA1 mutation in 1/57 (1.8%) children. A total of 19/57 (33.3%) children had more than one episode of acute pancreatitis during the considered period. A total of 10/57 (17.5%) children were obese. The clinical picture was dominated by abdominal pain, vomiting and jaundice. Complications were observed in 9/57 (15.8%) children: peripancreatic fluid collections (6/57—10.5%), pancreatic necrosis (4/57—7%), and pleural effusion and/or pseudocysts. *Conclusions:* The number of children diagnosed and treated with acute pancreatitis increased over time. The most frequent causes are genetic predispositions, infections and cholelithiasis. Acute pancreatitis should be considered in every case of abdominal pain, vomiting and jaundice in children. Complications with a severe course are also observed in the pediatric population with acute pancreatitis.

## 1. Introduction

Acute pancreatitis is a multifactorial, complicated inflammatory process that involves the organ and the tissues around it. It is caused by the premature activation of pancreatic proenzymes, which results in the proteolytic destruction of the organ’s tissue [1]. The incidence of acute pancreatitis in the pediatric population is not known, whereas in adults, it ranges from 10 to 80 per 100,000 per year. In recent years, there has been an increasing incidence of acute pancreatitis in the pediatric population [2]. In children, the most common causes of acute pancreatitis are abdominal trauma, infections (mostly viruses), systemic diseases, bile duct diseases or defects (e.g., cholelithiasis) and genetic mutations (SPINK, PRSS, CFTR). The clinical course of the disease varies widely from uncomplicated mild inflammation to severe pancreatitis with multiple organ failure and accompanying complications [3]. The mortality rate among adults can reach 6–10%. The clinical picture is dominated by abdominal pain located in the middle and upper abdomen, sometimes radiating around the body, along with nausea/vomiting and jaundice of biliary origin [4]. The diagnosis of acute pancreatitis is based on at least two out of three criteria: a clinical presentation with severe abdominal pain, elevated serum amylase/lipase levels exceeding three times the upper limit of normal and imaging findings (ultrasound, CT, MRI) consistent with acute pancreatitis. In some cases, pancreatic parenchymal necrosis and/or peripancreatic fat necrosis occurs and, less commonly, necrosis limited to the surrounding fatty tissue. Perfusion disturbances may persist for several days; hence, necrotic changes progress over time. The final extent of the changes may be underestimated if imaging studies are performed too early. In severe cases, the disease can lead to multiple organ failure (MOF) affecting the respiratory, circulatory and renal systems as well as acute systemic inflammatory response syndrome (SIRS). In the differential diagnosis of acute pancreatitis (AP), other causes of acute abdomen should be considered, such as bowel obstruction, gastrointestinal perforation, acute appendicitis, ischemic bowel disease, ectopic pregnancy and/or gallstones [5]. Therapeutic management depends on the cause of acute pancreatitis. Every patient with pancreatitis in the initial phase requires intensive intravenous hydration, correction of electrolyte imbalances and pain management. Biliary pancreatitis often necessitates an urgent ERCP procedure. Early initiation of nutritional therapy is important to prevent bacterial translocation and the spread of necrosis. Complications of acute pancreatitis in the pediatric population are not observed frequently; however, they can lead to life-threatening conditions (generalized inflammatory reactions, extensive fluid collections). A more common problem in the pediatric population is the recurrence of acute pancreatitis and the potential progression to a chronic form of the disease. The prevalence of acute recurrent pancreatitis is estimated to be 10–20% in the pediatric population. In long-term observations, children with recurrent AP require monitoring for the occurrence of exocrine and/or endocrine pancreatic insufficiency [1].

## 2. Materials and Methods

We retrospectively analyzed the history of 57 children hospitalized in the Department of Paediatrics, Medical University of Silesia in Katowice between 2019 and 2022 with a diagnosis of acute pancreatitis. The including criteria were an age below 18 years, formal agreement of the patient and/or legal guardian and at least one episode of AP. The excluding criteria were lack of a formal agreement of the patient and/or legal guardian, pregnancy and age above 18 years. The diagnosis was set based on ISPPIRE criteria, and its severity was evaluated using the PABS scale [6]. The following variables were taken into consideration: cause of pancreatitis, children’s age, sex, nutritional state, clinical symptoms, family history, duration of hospitalization, number of recurrences, results of laboratory tests and diagnostic imaging as well as complications. The authors divided the causes of acute pancreatitis into 4 groups: 1 = mutation and/or anatomical defect of pancreatic ducts (n = 14)—although genetic testing was run in all groups, 2 = idiopathic (n = 20), 3 = infectious (n = 6) and 4 = biliary (n = 14). The between-group differences were observed in terms of gender, BMI, number of AP episodes and duration of the hospitalization. The statistical analysis was performed using IBM SPSS 12.9 software. Qualitative variables were presented as frequency and percentages; quantitative variables were presented as mean with standard deviation (SD) or median with interquartile range (IQR). The distribution of quantitative variables was verified with the Shapiro–Wilk test, and homogeneity of variance was verified with Levene’s test. Between-group comparisons were performed with chi square, Mann–Whitney or t student tests, wherever applicable.

## 3. Results

### 3.1. General Characteristics of the Study Group

The analysis includes 57 children (age 2–18 years, average = 11 years), 29/57 (51%) boys and 28/57 (49%) girls, diagnosed with acute pancreatitis (AP). Most of the admitted children were aged above 10 years (32/57, 56.1%). There were no differences in age distribution between boys and girls (*p* = 0.307). A total of 19/57 (33.3%) children had a history of more than one AP episode (median = 1, IQR = 7), and 7/57 (12.3%) were diagnosed with chronic pancreatitis during several years of observation. The mean age of the first AP episode was 10.4 years (SD = 5.1, range 1–18 years). A total of 5/57 (8.8%) children had a familial history of pancreatic diseases. The average body mass index (BMI) of the examined patients was 50 percentiles (SD = 35.9, range 1–99 percentiles). Ten (17.5%) children were obese. The detailed characteristics of the study population are presented in Table 1.

### 3.2. Ethiopathogenesis

**Figure 1:**
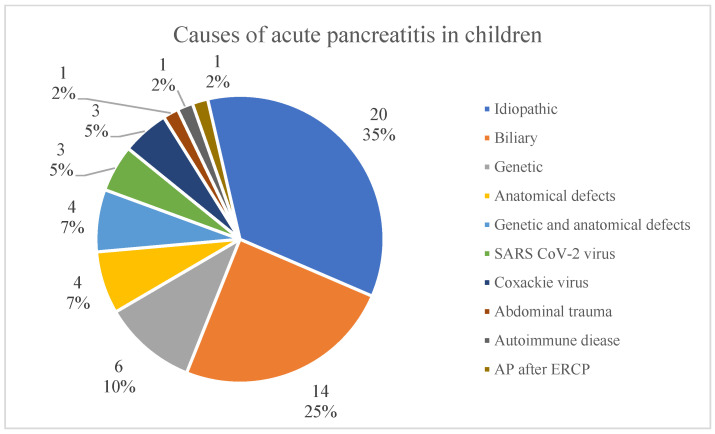
Causes of acute pancreatitis in children.

The most common causes of acute pancreatitis were biliary cholelithiasis 14/57 (24.6%), genetic 10/57 (17.5%) and anatomical abnormalities of pancreatic ducts (annular pancreas, bifid pancreas) 8/57 (14%), with 4/57 (7%) children suffering from both genetic and anatomical defects. In 20/57 (35.1%) children, idiopathic pancreatitis was diagnosed (none of these were caused by taking medicine). The genetically determined causes were the following: SPINK1 mutation in 5/57 (8.8%) children, PRSS1 mutation in 4/57 (7%) patients and CPA1 mutation in 1/57 children (1.8%). The causes of AP are presented in Figure 1.

**Figure 2:**
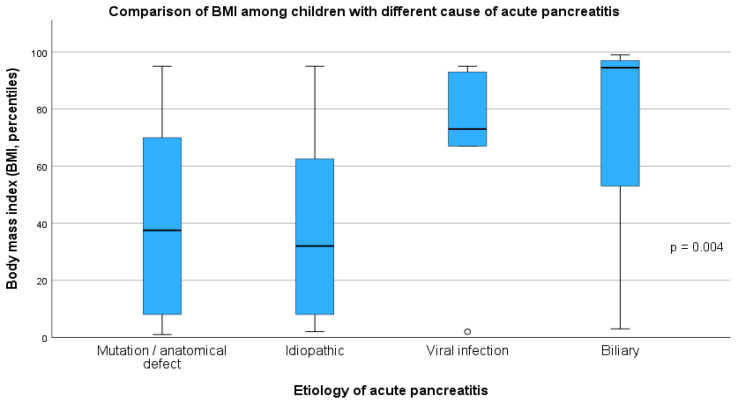
Comparison of body mass index (BMI) among groups of children with different acute pancreatitis etiology.

**Figure 3:**
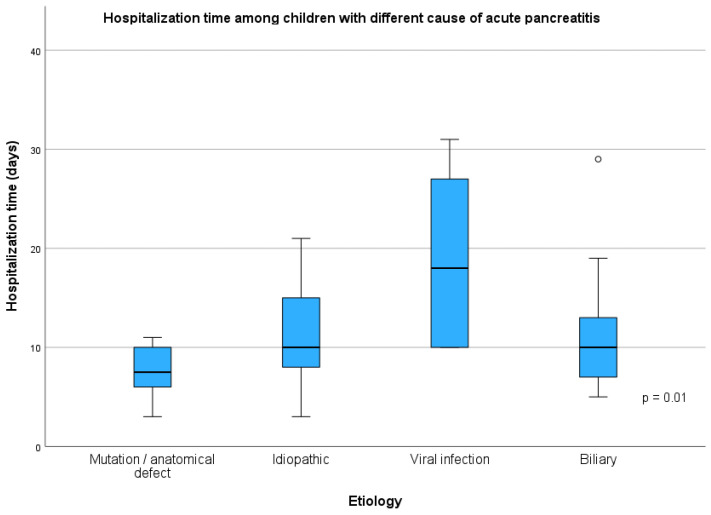
Hospitalization time among group of children with acute pancreatitis.

The most common causes of AP were included in the subgroup analysis. Analyzing the causes of acute pancreatitis, it was shown that there were more girls (12/14, 85.7%) with AP due to cholelithiasis than in the groups with other AP causes (*p* = 0.029), which was a statistically significant difference. Also, median BMI percentile was higher (*p* = 0.004) in patients with biliary etiology (95 pc, IQR = 46) than in patients with mutation/anatomical defect (50 pc, IQR = 57) and idiopathic AP (40 pc, IQR = 64) (Figure 2). A higher number of AP episodes were observed in children with mutation/anatomical defect (2, IQR = 3, *p* < 0.001). There was no difference in the severity of the AP course; however, the longest hospitalization time was required in patients with AP due to infectious causes (Coxackie or SARS-CoV-2 virus infection—the diagnosis was made based on the results of a swab test) (18 days, IQR = 18)—but the difference was significant (*p* = 0.006) only compared to children with mutation/anatomical defect (8 days, IQR = 4). The differences in duration of the hospitalization are presented in Figure 3. There was no significant difference in age distribution between analyzed groups; notably, we observed a lower age of the first AP episode in patients with mutation/anatomical defect and idiopathic etiology. The detailed results of the between-group comparisons are presented in Table 2.

### 3.3. Symptoms of Acute Pancreatits

Median duration of clinical symptoms of AP before the hospital admission was 2 days (IQR = 2, 1–7 days). The most common AP symptoms were abdominal pain 52/57 (91.2%), nausea and vomiting 31/57 (54.4%) as well as fever 13/57 (22.8%). Other symptoms were anxiety 9/57 (15.8%), loss of appetite 7/57 (2.3%), weakness 5/57 (8.8%), jaundice 4/57 (7%) and/or diarrhea 2/57 (3.5%).

### 3.4. Acute Pancreatitis Course and Treatment

**Figure 4:**
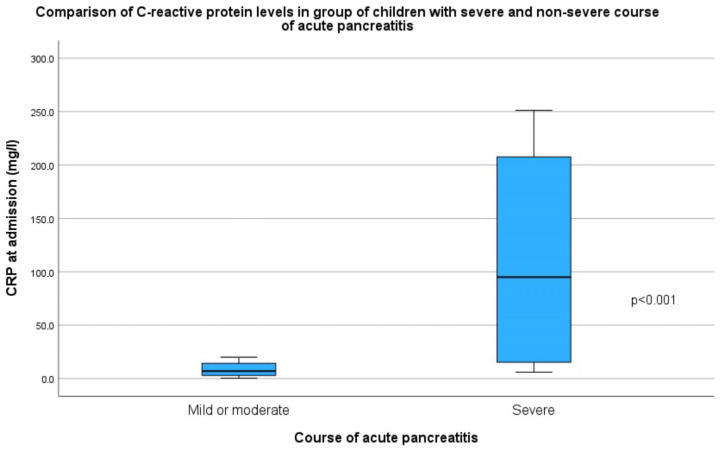
Comparison of C-reactive protein levels at admission in groups of children with different courses of acute pancreatitis.

Mean hospitalization time was 11 days (SD = 6.2 days, range 3–31 days). The majority of children were treated conservatively 49/57 (86%), 6/57 children (10.5%) required ERCP, and in 2/57 cases (3.5%), urgent cholecystectomy with bile duct exploration was performed. ERCP was performed in children with biliary AP (5/57). The whole group of children had abdominal ultrasound imaging. Abdominal CT was performed in 16/57 (28.1%) of patients, more frequently in children with a severe course of AP (*p* < 0.001). Parenteral feeding was administered in 14 cases (24.6%), predominantly in the severe course group (88.9% vs. 12.5%, *p* < 0.001). In children with mild AP, oral feeding was not discontinued; in those with a moderately severe course, it was necessary to initiate enteral nutrition (beyond the ligament of Treitz), and in children with a severe course, total parenteral nutrition was required. Complications occurred in 9/57 (15.8%) children, with a higher incidence in the severe course group (66.7% vs. 6.3%, *p* < 0.001), especially taking into account pancreatic necrosis (*p* < 0.001) and peripancreatic fluid collections (*p* = 0.004). A severe course of AP (according to the Ranson’s criteria) occurred in 9/57 (15.8%) patients. The most common cause of AP in this group was biliary 3/9 (33.3%). In the group of children with a severe course of AP, the median BMI was higher (*p* = 0.031) than that among the other children. A severe course of AP was associated with longer hospitalization (9 vs. 20 days, *p* < 0.001), higher C-reactive protein (CRP) levels at admission and after 48 h of hospitalization (*p* < 0.001 and *p* = 0.002, respectively) and higher white blood cell count (*p* = 0.016). The CRP level in groups of children with different courses of AP is presented in Figure 4.

The amylase and lipase activity were not associated with the course of AP. The detailed comparison of children with different courses of AP is presented in Table 3.

## 4. Discussion

The last few years have shown an increase in the incidence of AP in both adults and children. The global incidence has reached 30–40 cases per 100,000 population per year. However, in young patients, it is still considered a rather rare episode; globally, it is 10–15 cases per 100,000 children [1,7], while in 2013, it was estimated as 3.6–13.2 cases in 100,000 children [8]. In previous research led in our center, there were 51 children treated due to AP in the years 2004 and 2013 [9]. The age of acute pancreatitis occurrence differs from various regions and years of research. There was a retrospective study conducted in Taiwan, where the median age of children with AP during 1993 and 2008 was 8.2 ± 4.8 years, while in Wisconsin, in a similar time (1996–2003), the median age was 12.5 years [10,11]. Later, in the years 2013–2019, in the study run in Ohio, the median age was 13.4 years, and compared to our research, the median age was similar (11.0 years) [12]. There is also no difference in the sex of the patients. In our study, there were 51% boys, and the gender distribution is like that found in other studies [10,11,12]. When analyzing the etiology, the most common cause in our study was idiopathic. Compared to other research, the dominant causes of AP are idiopathic, systemic disease, cholelithiasis, trauma, drugs, genetic disorders and malignancies [13]. There were also several cases of AP described, caused by COVID-19, which also happened in our study in three cases [14]. However, AP seems to develop, and the cases are more and more frequent in children; there are similar causes described as the largest part of proportion. In research published in 2003 in the USA, trauma and systematic disease were described as the most common causes, and it seems like it has not changed because, in a similar study from 2020 in Australia, Coffey and Ooi distinguished multisystem disease, trauma and idiopathic AP as the most frequent [11,13]. When analyzing the other authors’ papers, there is also similarity in AP causes [15,16]. However, trauma seems like it is one of the most common causes of AP; it did not take as big a part in our studies as in the other authors’ studies because these patients were hospitalized in the surgical ward, which cases were not described in our study. Although trauma in our research was in the minority of causes, idiopathic, biliary—especially in overweight children—and genetic (SPINK1, PRSS or CPA1) causes are dominant, like in similar studies, but the percentage of its frequency diverges in different studies [11,13,17,18]. The symptoms of AP are unfortunately very often non-characteristic. In the pediatric population, the most frequent symptoms are abdominal pain, nausea and vomiting, so it is important to take into account AP when diagnosing [15,17]. In our study, abdominal pain was noticed in almost all of the patients, and it was commonly followed by vomiting. However, the third most common symptom noticed in our study group was fever. During the previous AP study led at our department, only a small percentage (3.8%) of children presented fever, even when not during infection, but it seems like a common symptom as well, described by Arvind and Mark or Abu-El-Haija et al. [9,17]. It is worth noting that the patients with AP on a genetic or malformation basis were most likely to develop recurrent AP; however, the hospitalization time was shorter compared to the patients with infectious AP. Moreover, only one child with AP due to an anatomical defect or mutation had a severe course of AP [16]. Colombian researchers showed in their paper that almost half of the patients with recurrent AP had anatomical disorders, while in a different study, besides anatomical disorders, genetic causes were most frequent in the children with recurrent AP [19,20,21]. The analysis of our patients’ nutritional status showed that the children with AP due to genetic and anatomical defects had a significantly lower BMI, which might be connected with recurrence of the disease, which eventually leads to pancreas failure. On the other hand, the highest BMI was noticed in the patients with biliary AP, which may have an etiopathogenetic connection. The same conclusions came up with the study of the scientists from the USA [22,23]. There has also been an interesting correlation found in other research run in Taiwan, where a correlation between high BMI level and the severity of AP has been shown, which is the same as in our study [10]. Well-known laboratory indicators of AP are serum amylase and lipase activities at least three times greater than the upper limit of normal. Both in our study and in other described cases, it was not specifically correlated with the severity of AP [11,17]. In our patients, the level of CRP was correlated with the severity of AP. Hamish et al. and Wei et al. also noticed the same correlation in their studies [24,25]. When performing a deeper analysis of the laboratory results, not only CRP but also other criteria like leukocytes might be predictive markers for the severity of AP [26]. Ultrasonography imaging is not only a quick and painless procedure but also a very useful tool when diagnosing AP. In our study, all patients had USG, which, in most cases, showed some characteristic changes like swelling, fluid presence or edema, and likewise, other studies proved the characteristic USG image [16]. It is a very helpful solution; however, it is different to perform when diagnosing the obese patient or when there is a lot of gases in the bowels [26]. USG is the most common way of image scanning the children when suspecting AP both in our research (100% of our patients had USG performed) and in other centers. However, the usage of USG differs from 40 to 100% in patients, depending on the country [27,28]. In some cases, when USG is not clear enough or/and during severe AP, CT was necessary, especially when necrosis is suspected [29]. The treatment depends on the severity of AP. If the case is mild, it is important to keep the patient hydrated and start the oral feeding as soon as possible, which also was enforced in our patients. If severe AP occurs, fluid resuscitation might be performed, and antibiotherapy might be run [17,21,29]. In the patients with biliary AP, ERCP was necessary to perform as soon as possible; then, a cholecystectomy needed to be performed. The procedure was urgent both in our study and in different papers described. The patients also needed to have the biliary obstruction removed [16,30]. A total of 6/57 of our patients needed ERCP: a total of 5/57 of them due to cholelithiasis and 1/57 due to anatomical defect. Additionally, 1/57 of AP cases were caused by ERCP. There is no specific severity scale of AP made for children, so worldwide, adult scales are used. In our study, due to the laboratory tests run in the patients, we used Ranson’s criteria at admission. A total of 48 of our patients (84%) were characterized as mild AP, which is quite the same as in other authors’ studies using the same criteria [31,32]. Fortunately, AP complications in children are not common. However, in our study, nine children had AP complications, predominantly pancreatic fluid collections, with a similar frequency to Trout et al. [33]. In four cases, pancreatic necrosis was described, which is a severe yet uncommon complication, which was also described in USA centers [17,34]. Children with mutations predisposing them to acute pancreatitis or an anatomical defect of the pancreas ducts are characterized by multiple, recurrent, mild episodes of disease. This predisposes them to a gradual loss of active pancreatic parenchyma and the development of chronic pancreatitis, which, along with frequent episodes of abdominal pain, contributes to poor weight in these children—they are characterized by a lower BMI compared to other children hospitalized for acute pancreatitis. Although the length of hospitalization is shorter, disease in these children is a serious health problem.

## 5. Conclusions

- Taking into account previous data from the gastroenterology decreased, the number of children diagnosed with acute pancreatitis increased over time.
- The most frequent causes are genetic predispositions, anatomical defects and cholelithiasis; however, in up to 35%, which is the majority of cases, the cause remains unidentified. The results show the necessity of further investigation on AP and finding the other reasons of morbidity.
- Acute pancreatitis should be considered in every case of stomachache, vomiting and jaundice in children.
- It seems that obesity and high inflammatory parameters on admission are risk factors for the severe course of the condition.

## 6. Limits

This research was led in one center, which also makes the studied group limited. The second limitation is the results were based on a retrospective analysis.

## Figures and Tables

**Table 1:** General characteristics of the study population.

Variable/Parameters	Total (n = 57)	Female (n = 28, 49%)	Male (n = 29, 51%)	*p*
**Age (years)**	11 (SD = 5.0, 2–18)	11 (SD = 4.8, 3–18)	10 (SD = 5.1, 2–18)	0.243
0–5 years	13 (22.8%)	4 (14.3%)	9 (31%)	0.307
6–10 years	12 (21.1%)	7 (25%)	5 (17.2%)	
>10 years	32 (56.1%)	17 (60.7%)	15 (51.7%)	
**BMI (pc)**	50 (SD = 35.9, 1–99)	53 (SD = 37.5, 2–99)	49 (34.6, 1–97)	0.431
Underweight (≤5 pc)	9 (15.8%)	5 (17.9%)	4 (13.8%)	0.693
Normal weight (6–84 pc)	33 (57.9%)	14 (50%)	19 (65.5%)	
Overweight (85–94 pc)	5 (8.8%)	3 (10.7%)	2 (6.9%)	
Obese (≧95 pc)	10 (17.5%)	6 (21.4%)	4 (13.8%)	
**Chronic pancreatitis**	7 (12.3%)	2 (7.1%)	5 (17.2%)	1.000
**Familial history of pancreatic disease**	5 (8.8%)	3 (10.7%)	2 (6.9%)	0.670
**More than 1 AP episode**	19 (33.3%)	9 (32.1%)	10 (34.5%)	0.851
1 episode	38 (66.7%)	19 (67.9%)	19 (65.5%)	0.501
2 episodes	10 (17.5%)	6 (21.4%)	4 (13.8%)	
>2 episodes	9 (15.8%)	3 (10.7%)	6 (20.7%)	
**Age of the first AP episode (years)**	10 (SD = 5.1, 1–18)	11 (SD = 5.4, 1–18)	10 (SD = 4.9, 2–18)	0.231

Quantitative variables with normal distribution are presented as mean, standard deviation (SD) and range; quantitative variables with not normal distribution are presented as medians with interquartile ranges (IQR) and range; qualitative variables are presented as frequencies with their percentages. BMI—body mass index, pc—percentiles, AP—acute pancreatitis.

**Table 2:** Comparison of groups of children with different AP causes.

Variable	Mutation and/or Anatomical Defects (1, n = 14)	Idiopathic (2, n = 20)	Infectious (3, n = 6)	Biliary (4, n = 14)	*p*	*p* Post Hoc
Female sex	6 (42.9%)	8 (40%)	2 (33.3%)	12 (85.7%)	**0.029**	1 vs. 2 = 0.868 1 vs. 3 = 1.000 **1 vs. 4 = 0.046** 2 vs. 3 = 1.000 **2 vs. 4 = 0.013** **3 vs. 4 = 0.037**
Age (years)	10 (8.1 years)	7 (11.0 years)	13 (4.3 years)	14 (5.5 years)	0.124	
Age of the first AP episode (years)	8 (8.0 years)	7 (12.0 years)	12 (3.6 years)	14 (4.8 years)	0.059	
BMI (pc)	50 (57.0 pc)	40 (64.0 pc)	73 (43.0 pc)	95 (46.0 pc)	**0.004**	1 vs. 2 = 1.000 1 vs. 3 = 1.000 **1 vs. 4 = 0.030 ** 2 vs. 3 = 0.671 **2 vs. 4 = 0.004** 3 vs. 4 = 1.000
Number of AP episodes	2 (3.0)	1 (1.0)	1 (2.0)	1 (0)	**<0.001**	**1 vs. 2 = 0.012** 1 vs. 3 = 0.530 **1 vs. 4 = 0.033 ** 2 vs. 3 = 0.415 vs. 4 = 1.000 3 vs. 4 = 1.000
Duration of hospitalization (days)	8 (4.0 days)	10 (8.0 days)	18 (18.0 days)	10 (6.0 days)	**0.010**	1 vs. 2 = 0.279 ** 1 vs. 3 = 0.006** 1 vs. 4 = 0.387 2 vs. 3 = 0.385 2 vs. 4 = 1.000 3 vs. 4 = 0.393
Severe AP course (number)	2 (14.3%)	2 (10%)	2 (33.3%)	3 (21.4%)	0.655	

Quantitative variables with normal distribution are presented as mean, standard deviation (SD) and range; quantitative variables with not normal distribution are presented as medians with interquartile ranges (IQR) and range; qualitative variables are presented as frequencies with their percentages. BMI—body mass index, pc—percentiles, AP—acute pancreatitis. Statistically significant results (*p* < 0.05) are shown in bold.

**Table 3:** The detailed comparison of children with different courses of AP.

	Total (57)	Severe Course (9/57, 15.8%)	Non-Severe Course (48/57, 84.2%)	*p*
Female sex	28/57 (49%)	4/9 (44.4%)	24/48 (50%)	1.000
Age (years)	11 (SD = 5.0)	12 (IQR = 6.5)	12 (IQR = 9.8)	1.000
Age of the first AP episode (years)	10 (SD = 5.1)	12 (IQR = 6.4)	11 (IQR = 9.3)	0.891
BMI (pc)	52 (IQR = 75)	93 (IQR = 46)	48 (IQR = 66)	**0.031**
Obesity	10/57 (17.5%)	4/9 (44.4%)	6/48 (12.5%)	**0.044**
Number of AP episodes	1 (IQR = 1)	1 (IQR = 0)	1 (IQR = 1)	1.000
Duration of hospitalization (days)	11 (SD = 6.2)	20 (IQR = 14)	9 (IQR = 4)	**<0.001**
**Etiology**	20/57 (25%)	2/9 (22.2%)	18/48 (37.5%)	0.379
- Idiopathic	14/57 (25%)	3/9 (33.3%)	11/48 (22.9%)	
- Biliary	14/57 (25%)	1/9 (11.1%)	13/48 (27.1%)	
- Genetic mutation or anatomical defect	3/57 (5%)	0/9	3/48 (6.3%)	
- Infectious SARS-CoV-2 or Coxackie virus	3/57 (5%)	2/9 (22.2%)	1/48 (2.1%)	
- Abdominal trauma	1/57 (2%)	1/9 (11.1%)	0/48 (0%)	
- Autoimmune disease	1/57 (2%)	0/9	1/48 (2.1%)	
- AP after ERCP	1/57 (2%)	0/9	1/48 (2.1%)	
Diagnostic imaging				
- USG	57/57 (100%)	9/9 (100%)	48/48 (100%)	
- Abdominal CT	16/57 (28.1%)	9/9 (100%)	7/48 (14.6%)	**<0.001**
- MR	17/57 (29.8%)	5/9 (55.6%)	12/48 (25%)	0.112
**Laboratory test results**				
Albumin (mg/dL)	41 (SD = 3.9)	39 (IQR = 5)	40 (IQR = 4.7)	0.183
ALT (U/L)	26 (IQR = 105)	31 (IQR = 112)	24 (IQR = 104)	0.829
AST (U/L)	35 (IQR = 49)	41 (IQR = 37)	35 (IQR = 71)	0.763
Amylase (U/L)	515 (IQR = 850)	302 (IQR = 719)	521 (IQR = 841.3)	0.538
Amylase after 48 h (U/L)	165 (IQR = 309)	104 (IQR = 677)	174 (IQR = 299)	0.732
Lipase (U/L)	772 (IQR = 1954)	473 (IQR = 821)	826 (IQR = 1958)	0.560
Bilirubin (U/L)	16 (IQR = 18.8)	15 (IQR = 17.8)	16 (IQR = 22.7)	0.861
AF (U/L)	172 (IQR = 77)	180 (IQR = 77.8)	158 (IQR = 99.5)	0.886
GGTP (U/L)	20 (IQR = 84)	28 (IQR = 76)	19 (IQR = 97.5)	0.913
CRP at admission (mg/L)	8 (IQR = 17.2)	119 (IQR = 201.3)	6 (IQR = 11)	**<0.001**
CRP after 48 h (mg/L)	10 (IQR = 67.5)	147 (IQR = 197.2)	8 (IQR = 18.5)	**0.002**
Highest CRP during hospital stay (mg/L)	20 (IQR = 93.4)	208 (IQR = 161.1)	14 (IQR = 61.1)	**<0.001**
WBC	9 (IQR = 4.1)	16 (SD = 6.9)	9 (SD = 3)	**0.016**
Triglycerides (mg/dL)	84 (IQR = 56)	95 (IQR = 24)	80 (IQR = 68)	0.832
Cholesterol (mg/dL)	144 (SD = 37.5)	151 (SD = 48.2)	142 (SD = 35.2)	0.678
**Treatment**				
Parenteral feeding	14/57 (24.6%)	8/9 (88.9%)	6/48 (12.5%)	**<0.001**
Cholecystectomy	2/57 (3.5%)	0/9 (0%)	2/48 (4.2%)	1.000
ERCP	6/57 (10.5%)	1/9 (11.1%)	5/48 (10.4%)	1.000
**Complications**	9/57 (15.8%)	6/9 (66.7%)	3/48 (6.3%)	**<0.001**
Necrosis	4/57 (7.0%)	4/9 (44.4%)	0/48 (0%)	**<0.001**
Peripancreatic fluid collections	6/57 (10.5%)	4/9 (44.4%)	2/48 (4.2%)	**0.004**
Pleural effusion	2/57 (3.5%)	1/9 (11.1%)	1/48 (2.1%)	0.298
Pancreatic pseudocyst	2/57 (3.5%)	2/9 (22.2%)	0/48 (0%)	**0.023**

## Data Availability

The original contributions presented in this study are included in the article. Further inquiries can be directed to the corresponding author.
